# Clinical and immunological differentiation of isolated IgG and combined IgG & IgM deficiencies from common variable immunodeficiency

**DOI:** 10.3389/fimmu.2026.1777332

**Published:** 2026-03-06

**Authors:** Yagmur Dogru, Faranaz Atschekzei, Damla Dogru, Torsten Witte, Georgios Sogkas

**Affiliations:** 1 Department of Rheumatology and Immunology, Hannover Medical School, Hannover, Germany; 2 Hannover Medical School, Cluster of Excellence RESIST (EXC 2155), Hannover, Germany

**Keywords:** bronchiectasis, combined IgG/IgM deficiency, common variable immunodeficiency, IgG deficiency, primary antibody deficiencies

## Abstract

**Objective:**

To assess the clinical relevance of diagnosing and classifying isolated IgG deficiency and combined IgG/IgM deficiency separately from CVID.

**Methods:**

In a retrospective cohort of patients with primary hypogammaglobulinemia, we evaluated and compared the clinical spectrum and immunological findings of patients with CVID, isolated IgG deficiency, and combined IgG/IgM deficiency.

**Results:**

In comparison to CVID, respiratory tract infections and gastrointestinal infections were less common in isolated IgG or combined IgG/IgM deficiency, while recurrent mucocutaneous herpes simplex virus reactivations were more common. With respect to immune dysregulation, splenomegaly and immune thrombocytopenic purpura were more frequently observed in CVID. Comparison of immunophenotypic data, revealed relatively lower class-switch memory B cell counts in CVID, while patients with IgG deficiency displayed lower transitional B cells. Survival analysis for these cohorts reveals a significant divergence in long-term outcomes, demonstrating that patients with CVID experience markedly lower overall survival rates.

**Conclusions:**

Comparison of CVID with isolated IgG deficiency or combined IgG/IgM deficiency revealed distinct immunophenotypic profiles, differences in both infectious and non-infectious manifestations, and markedly worse clinical outcomes in CVID. These findings suggest that CVID and unclassified antibody deficiencies – manifesting as isolated IgG deficiency or combined IgG/IgM deficiency – occupy different immunological niches. Consequently, our data support maintaining CVID as a distinct diagnostic entity, separate from IgG and IgG/IgM deficiencies, and highlight the need for tailored diagnostic approaches and follow-up strategies for these different forms of primary antibody deficiency.

## Introduction

Primary antibody deficiencies (PAD) are a heterogeneous group of disorders marked by recurrent infections, immune dysregulation and increased cancer risk (1). The most common symptomatic and well-characterized PAD is common variable immunodeficiency (CVID) (2). According to the European Society for Immunodeficiencies (ESID), CVID is defined by immunological criteria, including concurrently reduced IgA and IgG, low class-switched memory B cells and/or impaired specific antibody production (3). Other, less characterized PADs include IgG subclass deficiencies, which occur in patients with normal total IgG and preserved IgA and IgM levels (4). Studying the clinical spectrum of IgG subclass deficiencies, we previously demonstrated that combined IgG2 and IgG4 deficiency closely resembles common variable immunodeficiency (CVID), as – compared with other IgG subclass deficiencies – it is associated with more severe respiratory tract infections, bronchiectasis, and splenomegaly. Besides IgG subclass deficiencies, rare PADs include the isolated IgG deficiency (hereafter referred to in this manuscript as IgG deficiency) and the combined IgG and IgM deficiency (hereafter referred to as IgG/IgM deficiency). These disorders share overlapping clinical features with CVID, but have historically been separated from CVID, i.e. patients with dual IgA and IgG reduction. Especially patients with IgG/IgM deficiency were previously grouped under the broad diagnosis of CVID (5).

The clinical spectrum of IgG deficiency and IgG/IgM deficiencies remains poorly defined and the necessity of classifying them separately from CVID remains uncertain. To assess the clinical relevance of distinguishing IgG or IgG/IgM deficiency from CVID – which may influence patient management – we compared the clinical manifestations and treatment strategies of IgG and IgG/IgM deficiencies with those of CVID, evaluating both shared features and distinguishing characteristics.

## Materials and methods

### Study cohort

This retrospective cohort study included adult patients (age≥18 years) visiting the Immunology outpatient clinic of the Hannover University Hospital. All patients provided written informed consent and the study was approved by the Ethics Committee of the Hannover Medical School (approval 11223_BO_K_2024). Primary hypogammaglobulinemia was diagnosed based on persistently reduced serum IgG levels (<7 g/L), with or without concomitant IgA or IgM reduction, in the absence of secondary causes such as protein loss, medication effects or underlying malignancy (6). Clinical and laboratory data recorded between 10/2018 and 10/2025 were analyzed retrospectively. Diagnosis of PAD was based on the current ESID diagnostic criteria (available at https://esid.org/working-parties/registry-working-party/diagnosis-criteria/) (7). Immunological evidence of profound T cell deficiency, defined as CD4^+^ T cell counts <200/µl, naïve CD4^+^ T cell frequency <10% or impaired T cell proliferation in response to mitogens led to diagnosis of a combined immunodeficiency (CID). Clinical and immunological data were obtained from patients’ medical files. PAD-associated phenotypes were documented as described previously (8). In particular, those included recurrent upper and/or lower respiratory tract infections according to the national guidelines for the diagnosis of PIDs (https://register.awmf.org/de/leitlinien/detail/112-001) (9), bronchiectasis (computed tomography-confirmed), autoimmune cytopenias, such as autoimmune hemolytic anemia (AIHA), immune thrombocytopenic purpura (ITP), organ-specific autoimmunity (including vitiligo, psoriasis, insulin-dependent diabetes mellitus (IDDM), thyroidopathies, atrophic gastritis and arthritis), granulomatous disease, enteropathy and malignancies. Interstitial lung disease (ILD) was diagnosed based on typical computed tomography scan findings, in the absence of evidence for an infectious or alternative cause. Splenomegaly was defined as spleen enlargement ≥11 cm on palpation or ultrasound, including previous splenectomy of an enlarged spleen. Lymphadenopathy was detected on palpation, ultrasound, computed tomography or magnetic resonance imaging. Enteropathy included all cases of biopsy-proven non-infectious inflammatory bowel disease (IBD) (ulcerative colitis and Crohn’s disease), celiac disease, lymphocytic infiltration of the interepithelial mucous, the lamina propria and/or the submucosa as well as patients with chronic idiopathic diarrhea. Malignancies included hematologic and all other forms of cancer. Immunoglobulin levels were documented prior to the introduction of immunoglobulin replacement, while all considered immunoglobulin and lymphocyte count values were evaluated at least 6 months apart from treatment with immunosuppressive medications. Further, in all patients, lymphocyte count values were evaluated after the age of 18 years. Phenotypic analyses of lymphocytes from peripheral blood was performed as described previously (10). Briefly, peripheral blood mononuclear cells (PBMCs) were isolated from peripheral whole blood collected in sterile lithium heparin tubes. Phenotypic analyses were performed as multicolour immunofluorescence of PBMC, using directly labeled monoclonal antibodies. A total of 1x10^5^ to 2x10^6^ cells/well were incubated with murine monoclonal antibodies against the appropriate antigens at an optimal dilution for 20 min at 4°C. Nonspecific binding was eliminated by mixing the samples with a 1:5 solution of a commercial human IgG (Octagam, Octapharma). Samples were washed three times in PBS/BSA, and at least 10^4^ cells per appropriate gate were analyzed. The following antibodies (all purchased from Biolegend, if not otherwise stated) were used for this study: CD3 PerCP (BD Pharmingen), CD3 PE-Cy7, CD3 BUV563 (BD Biosciences), CD4 APC-Cy7, CD4 PerCP, CD8 PE, CD19 BV510, CD27 FITC, CD38 PE-Cy7, CD45 APC-Cy7, CD45RO BV421, CD45 BV785, CD45RA V500, CD56 BV421, IgD PE, IgM Alexa Fluor 647. Each flow cytometric analysis was controlled with appropriate isotype-matched antibodies. CD19^+^ cells in the lymphocyte gate were subdivided into the following subsets: naive B cells (IgD+, IgM+, CD27-), IgM^+^ memory/marginal zone B cells (CD27+, IgD+, IgM+), class-switched B cells (CD27+, IgM-, IgD-), CD21^low^ B cells (CD38 low, CD21 low). NK cells were gated as CD56^+^CD3^-^ cells. CD3^+^ T cells in the lymphocyte gate were subdivided into the following subsets: CD4^+^ T cells, CD8^+^ T cells, naive CD4^+^ T cells (CD4+, CD45RA+), CD4^+^ memory T cells (CD4+, CD45RO+), and follicular like CD4^+^ T cells (CD4+, CXCR5+). Gating strategy to measure studied lymphocyte subsets is shown in **Supplementary Figure 1**.

### Statistical analysis

For statistical calculation we used GraphPad prism 10 (GraphPad, La Jolla, USA). Descriptive statistics are reported as median and interquartile range (IQR) in case of continuous variables and as counts and percentages for dichotomous variables. Categorical variables were compared by the Yate’ s continuity corrected chi-squared test, which was employed to compare CVID with isolated IgG deficiency or combined IgG/IgM deficiency. Differences in continuous variants, including B cell and T cell counts were evaluated by ordinary one-way ANOVA and Tukey’s multiple comparison test, unless otherwise stated. Overall survival was estimated using the Kaplan-Meier method. Survival time was defined as age at death or age at last follow-up. The log-rank test was employed to compare survival curves between CVID and isolated IgG deficiency or combined IgG/IgM deficiency.

## Results

### Patients’ characteristics and survival rates

**Figure 1:**
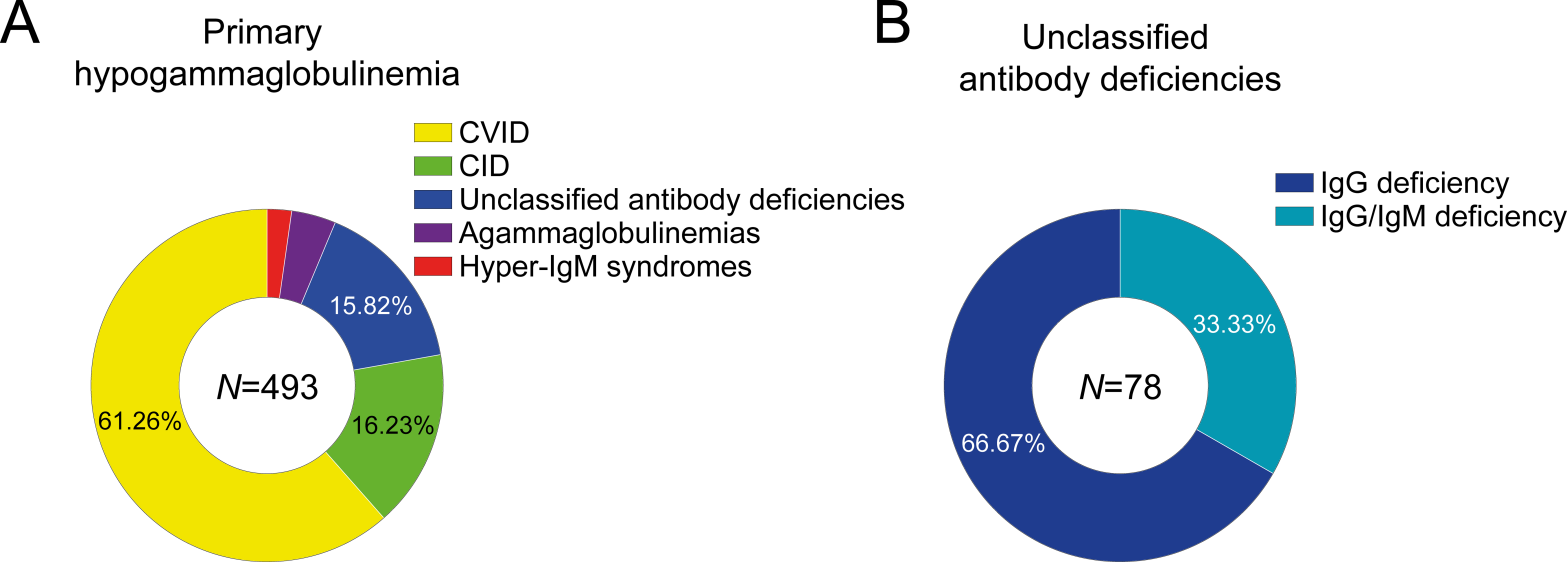
Primary immundeficiency disorders in 493 patients with primary hypogammaglobulinemia **(A)** and quotes of patients with IgG deficiency or IgG/IgM deficiency among 78 patients with unclassified antibody deficiencies **(B)** (CVID, common variable immunodeficiency; CID, combined immunodeficiency).

Of the 493 patients with primary hypogammaglobulinemia, who were screened, 302 (61.3%) were diagnosed with common variable immunodeficiency (CVID), in line with CVID being the most prevalent PAD (**Figure 1A**). CVID was followed in frequency by CID (80/493, 16.2%), unclassified antibody deficiencies (78/493, 15.8%), agammaglobulinemias (20/493, 4.1%) and hyper-IgM syndromes (11/493, 2.2%). The present study focused on comparing patients diagnosed with CVID to those with unclassified antibody deficiencies (altogether comprising 380 patients). These patients exhibited primary hypogammaglobulinemia but could not be assigned to any of the established diagnostic categories listed above. They either demonstrated isolated IgG reduction, i.e. IgG deficiency (52/493, 10.5%) or concomitantly reduced IgG and IgM (IgG/IgM deficiency; 26/493, 5.3%) (**Figure 1B**), without clinical or immunological evidence of profound T cell deficiency, which would otherwise support diagnosis of CID.

A female predominance was observed in both IgG deficiency and CVID, in contrast to patients with IgG/IgM deficiency. However, these differences did not reach statistical significance (**Table 1**). Patients with CVID tended to be younger than those with IgG deficiency or IgG/IgM deficiency, although comparative analysis of patients’ age revealed no significant differences (**Table 1**). Diagnosis of IgG deficiency was commonly established significantly later than that of CVID, which may reflect a milder phenotype and/or a later disease onset. Similar to the latter comparison, IgG/IgM deficiency was diagnosed at an older age than CVID (**Table 1**).

**Table 1:** Comparison of demographic data, infectious and noninfectious complications in patients with CVID and isolated IgG or IgG/IgM deficiency.

Patient characteristics	CVID (*N* = 302)	IgG deficiency (*N* = 52)	IgG/IgM deficiency (*N* = 26)	CVID vs. IgG deficiency			CVID vs. IgG/IgM deficiency		
				OR	95% CI	*p*-value^1^	OR	95% CI	*p*-value^1^
Demographic data									
Male sex, no. (%)	120 (39.74)	13 (25)	13 (50)	1.98	1.01-3.94	0.0613 (ns)	0.66	0.3-1.45	0.4152 (ns)
Age, years (IQR)	45.66 (32.4-59.13)	53.35 (34.53-59.18)	59.55 (34.85-65.83)	n.a.	n.a.	0.3847 (ns)	n.a.	n.a.	0.1018 (ns)
Age at diagnosis, years (IQR)	33.33 (22.19-44.97)	43.51 (28.75-51.99)	52.51 (30.98-62.43)	n.a.	n.a.	0.016 (*)	n.a.	n.a.	0.001 (***)
Deceased, no. (%)	15 (4.96)	0 (0)	1 (3.85)	+inf.	0.7-+inf.	0.2042 (ns)	1.31	0.2-14.33	0.826 (ns)
Infectious manifestations/complications									
Recurrent URTI, no. (%)	240 (79.47)	35 (67.31)	15 (57.69)	1.88	0.98-3.47	0.0775 (ns)	2.84	1.3-6.25	0.0206 (*)
Recurrent LRTI, no. (%)	211 (69.87)	25 (48.08)	15 (57.69)	2.5	1.36-4.44	0.0035 (**)	1.7	0.78-3.67	0.2864 (ns)
Bronchiectasis, no. (%)	63 (20.86)	8 (15.38)	6 (23.08)	1.45	0.67-3.09	0.4694 (ns)	0.88	0.36-2.2	0.9878 (ns)
Recurrent GI infections, no. (%)	67 (22.19)	4 (7.69)	1 (3.85)	3.42	1.25-9.13	0.0262 (*)	7.13	1.29-74.71	0.0498 (*)
Recurrent mucocutaneous HSV infections, no. (%)	17 (5.63)	12 (23.08)	6 (23.08)	0.18	0.08-0.4	<0.0001 (****)	0.2	0.07-0.57	0.0033 (**)
Herpes zoster, no. (%)	45 (14.9)	7 (13.46)	5 (19.23)	1.13	0.48-2.79	0.9532 (ns)	0.74	0.27-1.87	0.7603 (ns)
Noninfectious manifestations									
Autoimmunity, no. (%)	163 (53.97)	25 (48.08)	11 (42.31)	1.27	0.7-2.32	0.5244 (ns)	1.6	0.75-3.49	0.3478 (ns)
Thyroidopathy, no. (%)	55 (18.21)	8 (15.38)	3 (11.54)	1.23	0.56-2.62	0.7672 (ns)	1.71	0.56-5.55	0.5566 (ns)
Arthritis, no. (%)	36 (11.92)	12 (23.08)	2 (7.69)	0.45	0.22-0.93	0.051 (ns)	1.62	0.4-7.21	0.7436 (ns)
ITP, no. (%)	49 (16.23)	2 (3.85)	1 (3.85)	4.84	1.24-20.81	0.0328 (*)	4.84	0.86-51	0.1613 (ns)
AIHA, no. (%)	22 (7.28)	1 (1.92)	0 (0)	4.01	0.70-42.38	0.2525 (ns)	+inf.	0.54-+inf.	0.3095 (ns)
ILD, no. (%)	36 (11.92)	1 (1.92)	2 (7.69)	6.9	1.15-71.88	0.0535 (ns)	1.62	0.40-7.21	0.7436 (ns)
Enteropathy, no. (%)	79 (26.16)	15 (28.85)	4 (15.38)	0.87	0.45-1.63	0.814 (ns)	1.95	0.68-5.37	0.3283 (ns)
Psoriasis, no. (%)	18 (5.96)	5 (9.62)	2 (7.69)	0.6	0.21-1.53	0.4945 (ns)	0.76	0.19-3.48	0.9419 (ns)
Vitiligo, no. (%)	8 (2.64)	0 (0)	1 (3.85)	+inf.	0.38-+inf.	0.4952 (ns)	0.68	0.1-7.83	0.7895 (ns)
Atrophic gastritis, no. (%)	11 (3.64)	0 (0)	0 (0)	+inf.	0.58-+inf.	0.3343 (ns)	+inf.	0.28-+inf.	0.6728 (ns)
Lymphadenopathy, no. (%)	71 (23.5)	11 (21.15)	4 (15.38)	1.15	0.57-2.37	0.8462 (ns)	1.69	0.59-4.67	0.4819 (ns)
Splenomegaly, no. (%)	81 (26.73)	3 (5.77)	1 (3.85)	5.99	2.03-18.75	0.0018 (**)	9.16	1.67-95.82	0.0183 (*)
Cancer, no. (%)	48 (15.89)	5 (9.62)	1 (3.85)	1.78	0.7-4.32	0.3362 (ns)	4.72	0.84-49.78	0.1716 (ns)
Treatment									
Immunoglobulin replacement, no. (%)	231 (76.49)	29 (55.77)	13 (50)	2.58	1.41-4.81	0.0031 (**)	3.25	1.44-7.29	0.0062 (**)
Antibiotic prophylaxis, no. (%)	73 (24.17)	8 (15.38)	5 (19.23)	1.75	0.82-3.73	0.2245 (ns)	1.34	0.51-3.35	0.743 (ns)
Antiviral prophylaxis, no. (%)	5 (1.66)	2 (3.85)	2 (7.69)	0.42	0.09-2.17	0.6109 (ns)	0.2	0.04-1.07	0.1813 (ns)
Immunomodulatory treatment, no. (%)	40 (13.24)	14 (26. 92)	3 (11.54)	0.41	0.2-0.85	0.0201 (*)	1.17	0.37-3.84	0.9558 (ns)

AIHA, autoimmune hemolytic anemia; CI, confidence interval; CVID, common variable immunodeficiency; GI, gastrointestinal; ILD, interstitial lung disease; inf., infinity; IQR, interquartile range; ITP, immune thrombocytopenic purpura; LRTI, lower respiratory tract infections; N, total number; n.a., not applicable; n.s. not significant; no., number; OR, odds ratio; URTI, upper respiratory tract infections.

^1^p < 0.05 *; p < 0.01 **; p < 0.001 ***; p < 0.0001 ****.

**Figure 2:**
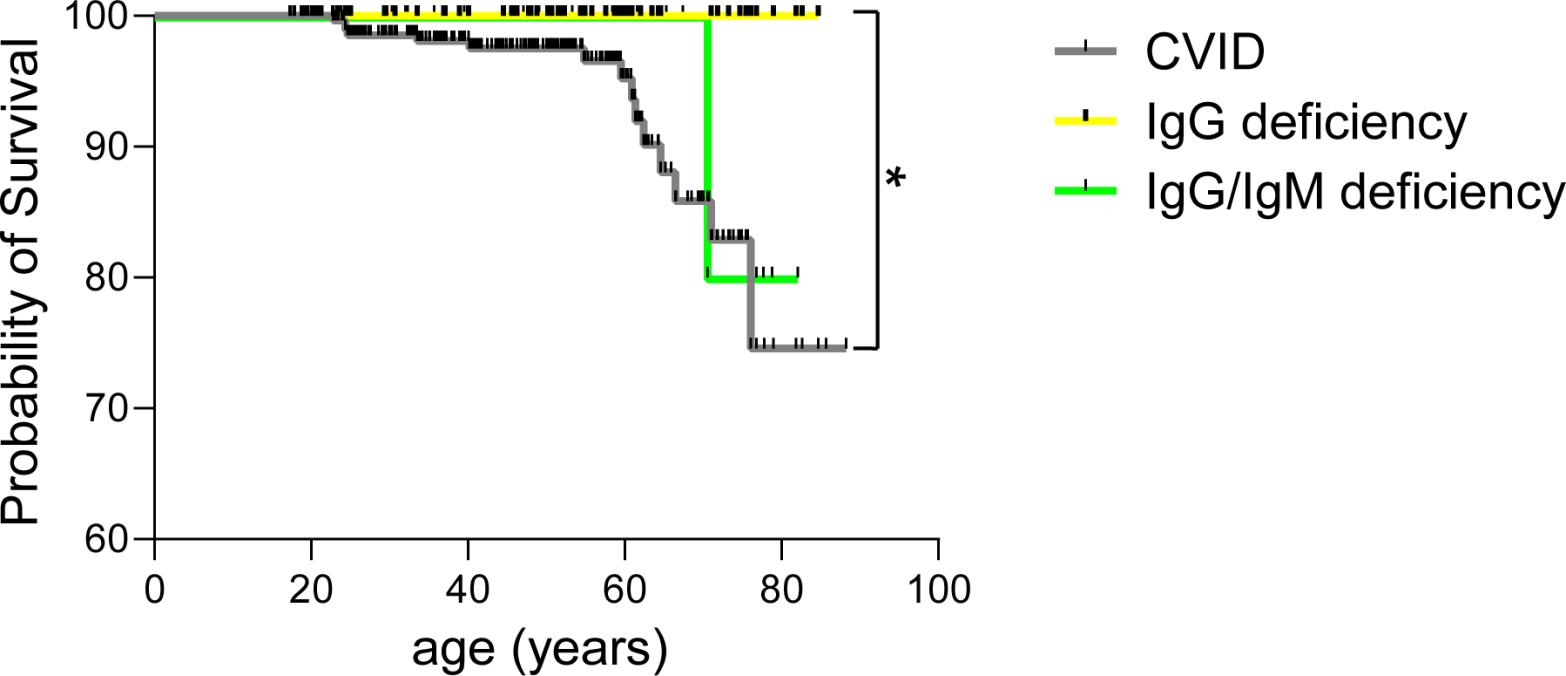
Comparison of age-dependent overall survival in patients with common variable immunodeficiency (CVID), IgG deficiency and IgG/IgM deficiency (*p* < 0.05 *).

Altogether 16 patients out of the 380 (4.2%) included in the present study were reported dead (**Table 1**). Most of them (15/16, 93.7%) were diagnosed with CVID and a single patient (1/16, 6.3%) with IgG/IgM deficiency (**Table 1**). The cause of death was known in all but one patient. The causes of death in case of CVID included infections (6/15, 40%), malignancies (5/15, 33.3%), liver failure (2/15, 13.3%) and transfusion-related acute lung injury (1/15, 6.7%). The patient with IgG/IgM deficiency died of acute myocardial infarction. Differences in the overall rate of malignancies were not significant between CVID and IgG or IgG/IgM deficiency. Nevertheless, types of cancer were different (**Supplementary Table 1**). While lymphomas, non-melanoma skin cancer and gastric cancer were relatively common in CVID, none of these cancer types were diagnosed in IgG deficiency. Fatal infections were heterogeneous, including bacterial sepsis in the context of neutropenia, pneumonia and pyelonephritis with septic course. A single patient died of COVID-19, while an additional patient of *Pneumocystis jirovecii* pneumonia. Interestingly immunological investigations at diagnosis of primary immunodeficiency in the latter two patients revealed no evidence of a combined immunodeficiency. The former patient was under low-dose prednisolone and abatacept due to granulomatous and lymphocytic interstitial lung disease (GLILD). To assess the impact of individual primary antibody deficiencies (PADs) on long-term outcomes, Kaplan-Meier survival analyses were performed (**Figure 2**). Patients with isolated IgG deficiency demonstrated a 100% survival probability throughout the documented follow-up period. In contrast, patients with common variable immunodeficiency (CVID) exhibited a progressive decline in survival probability beginning after 20 years of age, with a more pronounced decrease after the age of 60. Patients with combined IgG/IgM deficiency followed a survival trajectory comparable to that of the IgG deficiency group until approximately the age of 70.

### Clinical spectrum of IgG deficiency as compared to CVID

In line with the comparatively later disease onset and presumed milder phenotype of IgG deficiency, a history of LRTIs was significantly more frequent in patients with CVID (**Table 1**). In contrast, recurrent mucocutaneous herpes simplex reactivations occurred more commonly in IgG deficiency. Patients with IgG deficiency also showed a lower prevalence of recurrent gastrointestinal infections, possibly indicating a protective effect of preserved IgA in contrast to CVID. Overall, immune dysregulation in IgG deficiency resembled that observed in CVID, including manifestations of autoimmunity and benign lymphoproliferation. However, immune cytopenias, particularly ITP, and splenomegaly were significantly more common in CVID. Aside from the comparatively lower frequency of LRTIs, patients with isolated IgG deficiency were also less frequently treated with immunoglobulin replacement therapy, further suggesting a milder susceptibility to infections.

### Clinical spectrum of IgG/IgM deficiency as compared to CVID

While upper respiratory tract infections were more frequently documented in CVID, recurrent mucocutaneous herpes reactivations were more common in patients with IgG/IgM deficiency (**Table 1**). Immune dysregulation in IgG/IgM deficiency resembled that seen in CVID, including occurrences of arthritis and interstitial lung disease. However, ITP was relatively less frequent in IgG/IgM deficiency and no cases of autoimmune hemolytic anemia were observed in this patient group, while splenomegaly remained more frequent in CVID. Consistent with the pattern observed in isolated IgG deficiency, patients with IgG/IgM deficiency were less frequently treated with immunoglobulin replacement therapy, suggesting an overall milder susceptibility to infections.

### Comparison of immunoglobulin levels and immunophenotypic profiles in IgG deficiency and CVID

**Figure 3:**
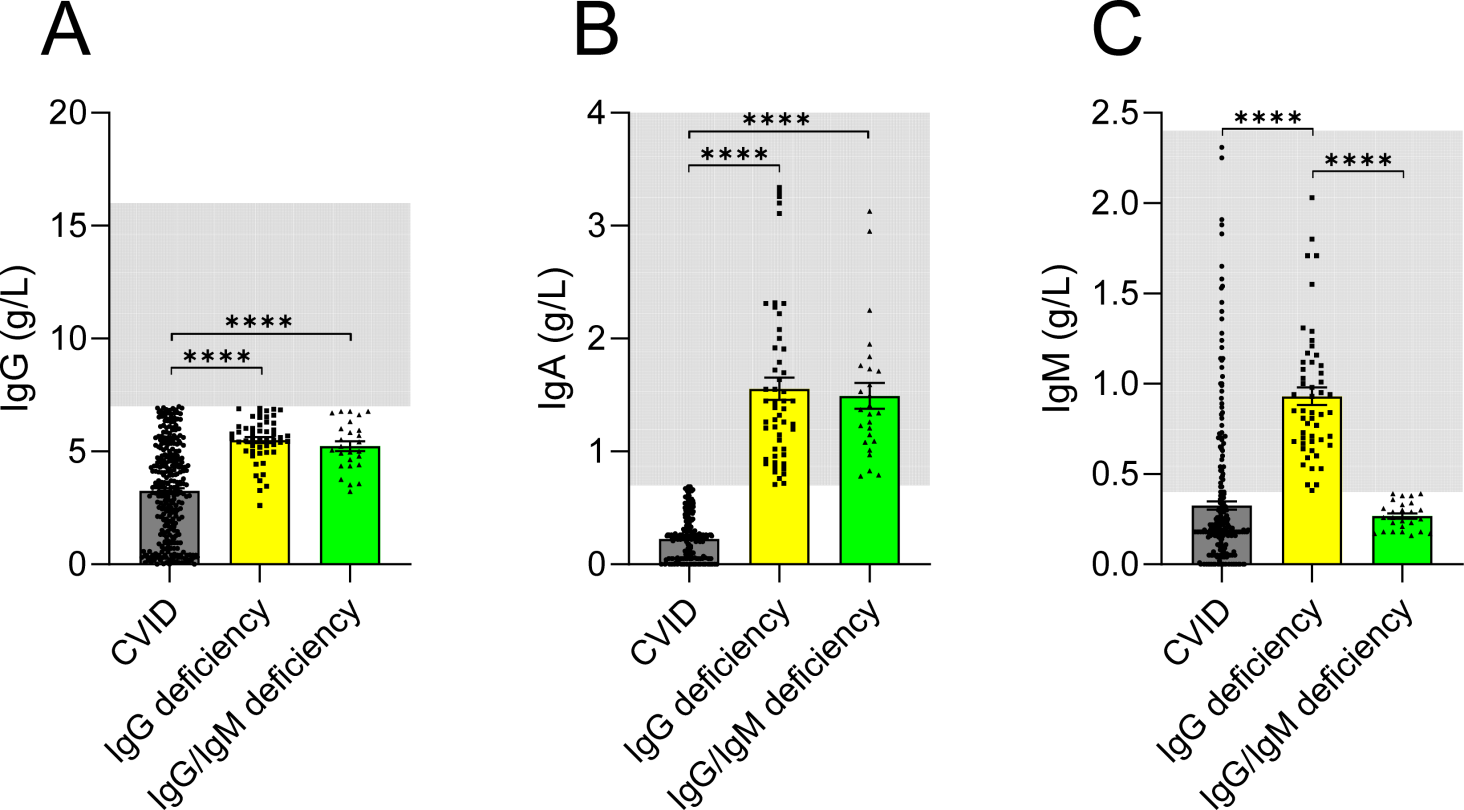
IgG **(A)**, IgA **(B)**, and IgM **(C)** levels in common variable immunodeficiency (CVID), IgG deficiency and IgG/IgM deficiency. Light gray shading indicates the normal reference range for adults, which applies to all patients except eight individuals (7 with CVID and 1 with IgG deficiency), whose values were measured prior to the age of 18 years (p < 0.0001 ****).

Patients with IgG deficiency displayed substantially higher serum IgG levels compared with CVID patients (IgG deficiency: 5.59 g/l (5.01-6.16) vs. CVID: 3.34 g/l (1.29-4.91)) (**Figure 3A**). By definition, IgA and IgM levels were normal in IgG deficiency and consequently higher than in CVID patients (**Figures 3B, C**). As expected from the relatively high proportion of IgG1 within total IgG, reduced IgG1 levels were detected in 47 of 48 patients with IgG deficiency (97.9%) for whom measurements of IgG subclasses were available (**Supplementary Figure 2A**). In 10 of 48 patients (20.8%), IgG1 was the only reduced subclass, while all other IgG subclasses remained within the normal range (**Supplementary Figure 2B**). Although, in theory, an isolated reduction in any IgG subclass could account for decreased total IgG concentrations (i.e., hypogammaglobulinemia), no patient exhibited an isolated reduction in any subclass other than IgG1.

**Figure 4:**
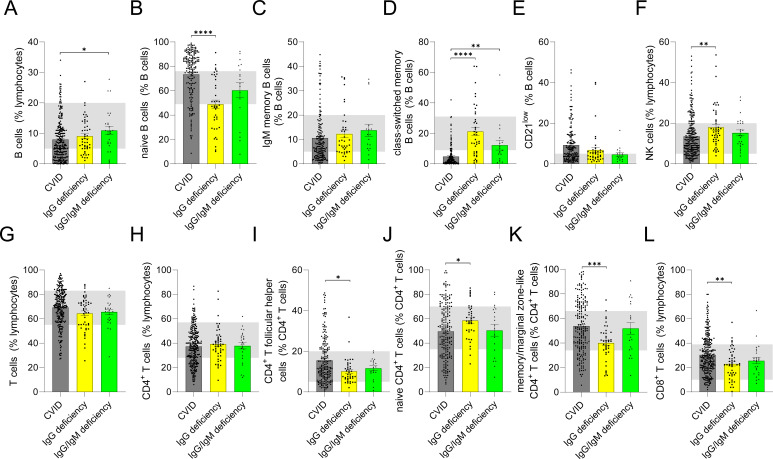
Comparison of lymphocyte subsets in common variable immunodeficiency (CVID), IgG deficiency and IgG/IgM deficiency. Following lymphocyte subsets are shown: B cells **(A)** and B cell subsets **(B–G)**, NK cells **(H)** as well as T cells **(I)** and T cell subsets **(J–N)**. Light gray shading indicates the normal reference range for adults (*p* < 0.05 *; *p* < 0.01 **; *p* < 0.001 ***; p < 0.0001 ****).

A reduced proportion of class-switched memory B cells is a well-established immunophenotypic hallmark of CVID, reflecting a defect in germinal center-dependent B cell differentiation (11, 12). In line with this, patients with CVID displayed markedly lower percentages of class-switched memory B cells than individuals with isolated IgG deficiency [(IgG deficiency: 17.5% (7.35-31.93) vs. CVID: 2.4% (0.83-5.9)], whereas naïve B cell frequencies were lower in isolated IgG deficiency [(IgG deficiency: 47.5% (31.45-71.08) vs. CVID: 79.85% (61.68-88.23)] (**Figure 4**). Taken together, these immunophenotypic differences support the presence of distinct abnormalities in B cell maturation pathways in CVID versus isolated IgG deficiency. With respect to CD4^+^ T cells, IgG deficiency was characterized by relatively higher proportions of naïve cells at the expense of memory subsets. Increased frequencies of circulating T follicular helper cells have previously been reported in CVID, indicating dysregulated germinal center activity (13). Accordingly, patients with CVID in our cohort exhibited significantly higher frequencies of circulating T follicular helper cells than those with isolated IgG deficiency. Finally, individuals with IgG deficiency showed a lower relative frequency of CD8^+^ T cells and a higher relative frequency of NK cells compared with CVID patients.

While the percentages of B cell or T cell subsets better reflect the relative composition, maturation, and differentiation within the respective cell compartment, absolute cell counts provide critical insight into B or T cell development and maturation (14, 15). Analyses of absolute cell counts were largely concordant with the relative frequency data (**Supplementary Table 1**). In particular, class-switched memory B cells were reduced in CVID compared with IgG deficiency also at the level of absolute counts. Similarly, reductions in NK cells and naïve CD4^+^ T cells observed in CVID compared with IgG deficiency were confirmed when evaluated as absolute cell numbers. Given the overall lower total absolute B cell count in CVID compared with IgG deficiency, analysis of absolute B cell subset numbers revealed significantly reduced IgM memory B cells in CVID. In contrast, although the relative frequency of naïve B cells was significantly increased in CVID compared with IgG deficiency, their absolute counts were similar. Furthermore, absolute CD4^+^ T cell counts were significantly lower in CVID compared with IgG deficiency.

### Comparison of immunoglobulin levels and immunophenotypic profiles in IgG/IgM deficiency and CVID

Similar to isolated IgG deficiency, patients with IgG/IgM deficiency displayed higher serum IgG levels than those with CVID [IgG/IgM deficiency: 5.15 g/l (4.40-6.09) vs. CVID: 3.34 g/l (1.29-4.91)] (**Figure 3A**). As expected from the definition of this phenotype, IgM levels were significantly lower in IgG/IgM deficiency than in CVID (**Figures 3B, C**). Immunophenotyping showed that, analogous to isolated IgG deficiency, patients with IgG/IgM deficiency had higher proportions of class-switched memory B cells compared with CVID [IgG/IgM deficiency: 8.65% (3.23-14.95) vs. CVID: 2.4% (0.83-5.9)] (**Figure 4**). The later finding was recapitulated by the measurement of absolute numbers of class-switched memory B cells (**Supplementary Table 1**). Similar to the comparison between CVID and IgG deficiency, CVID patients showed significantly lower absolute B cell counts, which resulted in significantly reduced absolute numbers of IgM memory B cells. In contrast, the relative frequencies of IgM memory B cells did not differ between groups, reflecting the overall reduction in total B cell numbers rather than a selective loss of this subset. For the other B cell subsets, as well as NK and T cell populations, no significant differences were observed between CVID and IgG/IgM deficiency.

## Discussion

In contrast to the well-characterized clinical and immunological spectrum of CVID, the phenotypic features of IgG deficiency and combined IgG/IgM deficiency remain incompletely defined. Our study demonstrates that, although these PADs share considerable overlap in terms of infectious susceptibility and immune dysregulation, important differences persist. Notably, patients with CVID experienced a higher frequency of recurrent gastrointestinal and lower respiratory tract infections compared with those with IgG deficiency. This observation suggests a more profound or qualitatively distinct immune defect in CVID, which may be partly attributable to the IgA deficiency intrinsic to CVID, leading to impaired mucosal defense mechanisms (16, 17). To date, only two studies have directly compared infectious manifestations between CVID and IgG deficiency (18, 19). Filion et al. reported comparable rates of pneumonia and sinusitis between the two conditions (18), whereas Gerek et al., in line with our findings, observed a higher frequency of pneumonia among CVID patients (19). In contrast to our results, however, both studies described nearly two-fold higher rates of bronchiectasis in CVID compared with IgG deficiency. Another comparative study, identified no difference with respect to bronchiectasis between CVID and a group of patients with idiopathic primary hypogammaglobulinemia, the majority of whom would fall under IgG deficiency (20). In our cohort, similar rates of bronchiectasis between groups may be explained by a delayed diagnosis of primary immunodeficiency in patients with IgG deficiency, as well as the higher median age in this group, both of which may contribute to cumulative structural lung damage. Beyond infectious manifestations, we also observed differences in immune dysregulation. Consistent with previous reports, splenomegaly and ITP were significantly more prevalent in patients with CVID in our cohort (18, 19).

In line with the aforementioned clinical discrepancies suggesting differential immune dysfunction, B cell immunophenotyping revealed distinct patterns between patients with IgG deficiency and those with CVID. In particular, patients with CVID exhibited a marked reduction in class-switched memory B cells, a well-established immunological hallmark of CVID that reflects defective germinal center reactions, impaired T cell-dependent B cell differentiation (21). With respect to T cells, the finding of relatively reduced CD8^+^ T cells in IgG deficiency comes in line with the study by Filion et al. (18). Further, the finding of elevated circulating follicular T cells in CVID as compared to IgG deficiency, is consistent with dysregulated germinal center responses in CVID (22). Overall, divergent B and T cell phenotypes indicate that isolated IgG deficiency and CVID likely represent immunopathogenetically distinct entities. Whereas IgG deficiency may predominantly involve defects at earlier stages of B cell development or homeostasis, CVID is characterized by failures in antigen-driven maturation and memory formation.

While patients with IgG/IgM deficiency were previously classified as CVID based on the ESID/Pan-American Group for Immunodeficiency (PAGID) classification criteria, representing a rarer disease subset (5, 23), recent classification criteria defined IgA reduction as a prerequisite for diagnosing CVID, separating IgG/IgM deficiency from CVID. This clinical spectrum of IgG/IgM deficiency had not been separately studied before. Here we identified a comparative higher rate of respiratory tract infections and gastrointestinal infections, as compared to CVID. The latter findings together with the latter diagnosis, similar to the case of IgG deficiency, are suggestive of an overall milder humoral immunodeficiency. Furthermore, the relatively higher quotes of class-switched memory B cells suggest a differential immunopathogenesis of B cell dysfunction.

Despite patients with IgG deficiency and IgG/IgM deficiency being relatively older than those with CVID, fatal outcomes were observed almost exclusively in the CVID cohort, while the single death documented outside the CVID group was not clearly attributable to immunological dysfunction. In line with previous reports, mortality in CVID was primarily associated with severe infections and malignancies (24). With respect to malignancies, it is noteworthy that no case of CVID-related cancers, i.e. lymphoma or gastric cancer, were detected in case of patients with IgG deficiency.

Our study has several limitations, primarily related to its retrospective design. In addition, the relatively small number of patients with IgG deficiency and IgG/IgM deficiency, limited detailed phenotypic analyses and may have reduced the power to detect statistically significant differences. This limitation underscores the need for multicenter studies including larger patient cohorts. Furthermore, regarding the immunophenotypic analyses, we acknowledge that the assessment of additional lymphocyte subsets could have provided deeper insight into the differential pathogenesis of the PADs studied. In addition, a more comprehensive characterization of the B cell compartment - including isotype expression of memory B cells and plasma cells - might have yielded additional prognostic information and helped to explain the marked phenotypic heterogeneity observed among these PADs (25). Another limitation is that all patients were recruited from a specialized outpatient clinic focusing on immunodeficiency, which may have led to an overestimation of infectious manifestations, especially in case of patients with unclassified antibody deficiencies. Nevertheless, the comparative evaluation of phenotypes and treatment strategies within a single center – using uniform diagnostic and documentation procedures – represents a key strength of the present study.

Genetic testing has enabled the identification of monogenic defects in patients with primary immunodeficiency disorders, including PADs, providing key insights into their clinical and immunophenotypic heterogeneity (26). A limitation of this work is the lack of genetic characterization of studied PAD cohort. While our primary objective was to compare clinically defined PAD subgroups, we recognize that underlying monogenic variants may influence individual disease trajectories. Nevertheless, several studies in cohorts with sporadic immunodeficiency have reported relatively modest diagnostic yields from next-generation sequencing approaches, including whole-genome sequencing, typically around 10-20% (27–29). Given our cohort’s characteristics, consisting mainly of patients with late-onset PADs and without features suggestive of combined immunodeficiency, the yield would likely be even lower. Considering this and the current limited availability of genetic testing in routine clinical practice, our findings regarding the differential phenotypic, immunological, and prognostic features of the studied PADs remain clinically meaningful.

Although primary antibody deficiencies share a partially overlapping clinical spectrum, comparative analysis of CVID versus IgG deficiency and IgG/IgM deficiency revealed distinct immunophenotypic signatures, differences in both infectious and non-infectious manifestations, and substantially worse clinical outcomes in CVID. Together, these findings indicate that CVID and unclassified antibody deficiencies – manifesting as isolated IgG deficiency or combined IgG/IgM deficiency – occupy different immunological niches rather than representing variations along a single disease continuum. In the context of the ongoing debate regarding PAD classification, our data support maintaining CVID as a distinct diagnostic entity separate from IgG and IgG/IgM deficiencies. This distinction has direct clinical implications and argues for diagnosis-specific follow-up strategies, particularly with regard to surveillance for malignancy and immune dysregulation such as autoimmunity.

## Data Availability

The raw data supporting the conclusions of this article will be made available by the authors, without undue reservation.
