# Supplementary material for: Clinical and immunological differentiation of isolated IgG and combined IgG & IgM deficiencies from common variable immunodeficiency
Source: Front Immunol. 2026 Mar 6;17:1777332. doi: 10.3389/fimmu.2026.1777332 (PMC13002413; doi:10.3389/fimmu.2026.1777332)
Supplement: Supplementary file 1 [file DataSheet1.pdf]

Supplement

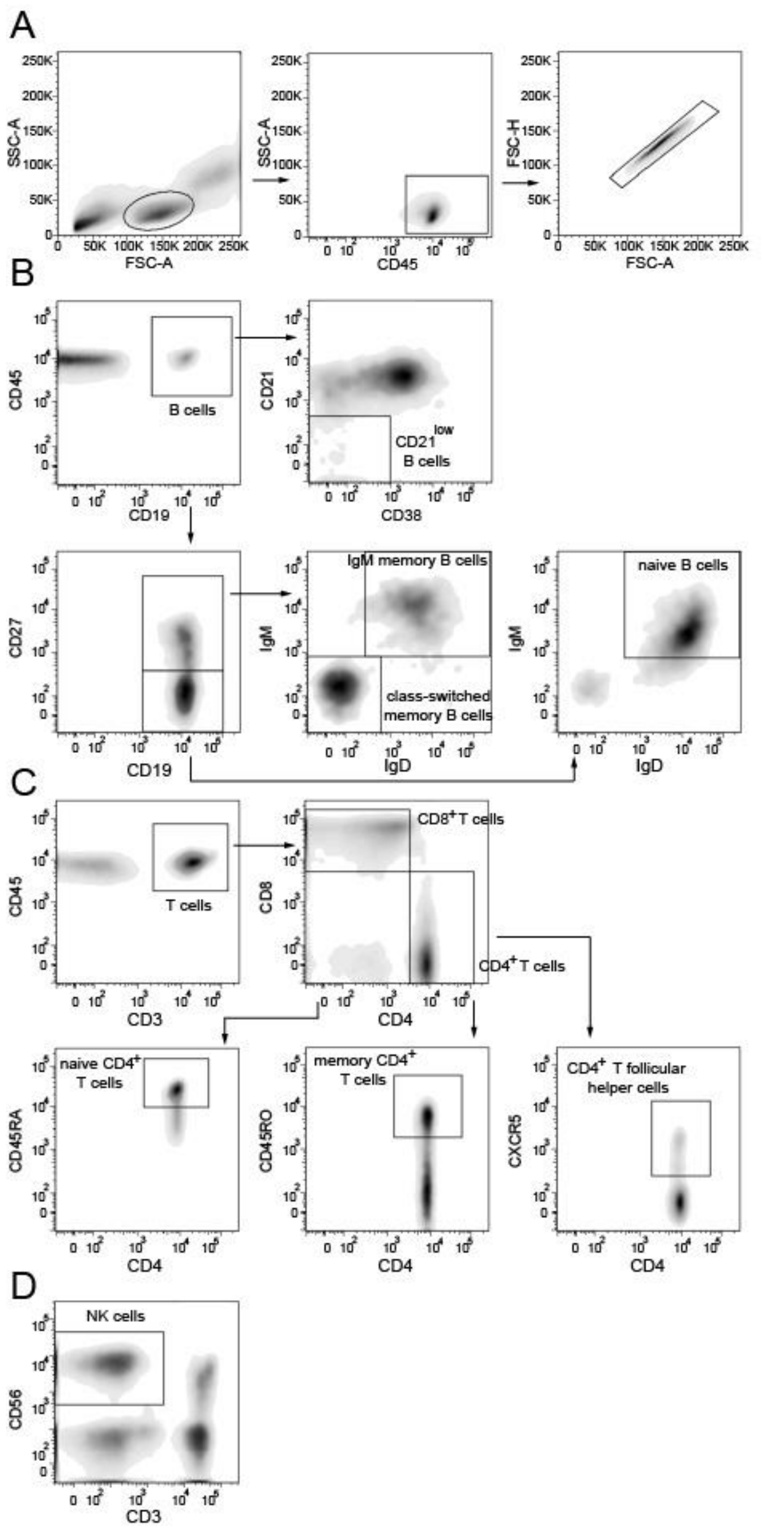

Suppl. Figure 1: Representative flow cytometry plots showing strategy to measure studied lymphocyte subsets. After gating on lymphocytes based on SSC, FSC and CD45 expression cells as well as doublet exclusion (A), we analyzed B cells (B), T cells (C) and NK cells (D) as indicated.

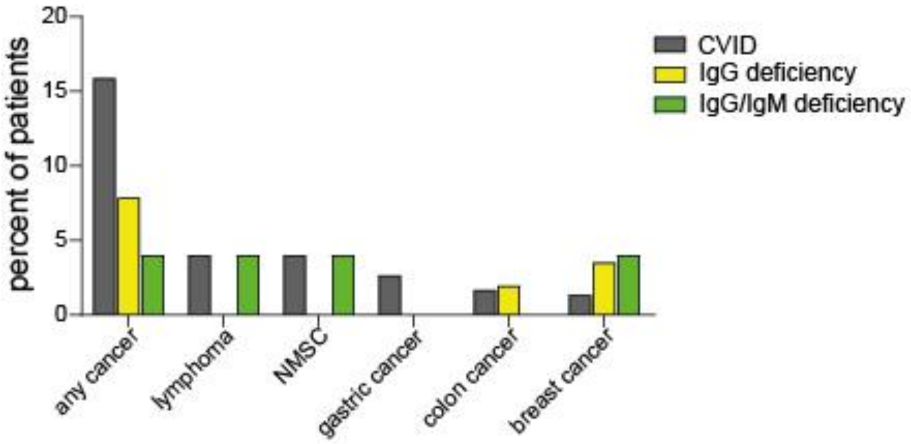

Suppl. Figure 2: Malignancies in patients with common variable immunodeficiency (CVID), IgG deficiency and IgG/IgM deficiency (MMSC, non-melanoma skin cancer)

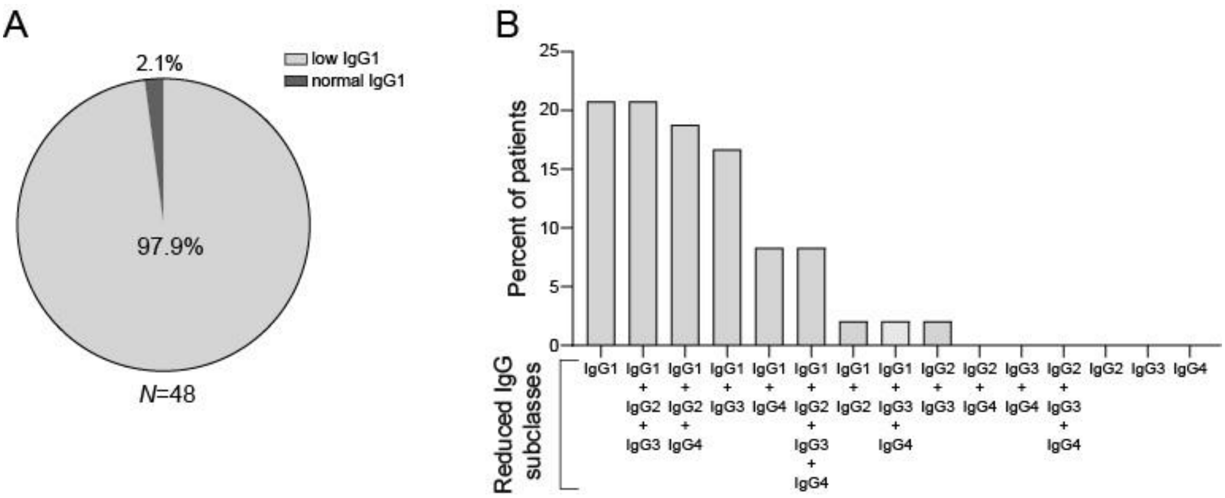

Suppl. Figure 3: IgG subclasses in 52 patients with IgG deficiency. Quote of patients with reduced IgG1 (A) and distribution of patients across different patterns of reduced IgG subclasses (B)

Suppl. Table 1: Comparison of absolute counts of lymphocytes and lymphocyte subsets in patients with CVID and isolated IgG or IgG/IgM deficiency

**Absolute cells counts (cells/µl)**	**CVID (*N*=302)**	**Isolated IgG deficiency (*N*=52)**	**Combined IgG & IgM deficiency (*N*=26)**	**Reference range**	**CVID vs. IgG deficiency *p*-value[1]**	**CVID vs. IgG/IgM deficiency *p*-value[1]**
**Lymphocytes, median (IQR)**	1,433 (982-1,970)	1,600 (1,189-2,270)	1,620 (1,095-2,359)	1,100-4,500	0.1395 (ns)	0.707 (ns)
**B cells, median (IQR)**	86.5 (36.7-160)	118 (71.5-213.5)	150.5 (102.8-150.5)	90-500	0.039 (*)	0.011 (*)
**Naïve B cells, median (IQR)**	68 (32-124)	53 (31-101.8)	98 (32.7-184)	50-300	>0.9999 (ns)	>0.9999 (ns)
**IgM memory/marginal zone-like B cells, median (IQR)**	6 (3-18)	12.5 (5-31.7)	18.5 (9.7-42.7)	5-50	0.0185 (*)	0.0108 (*)
**Class-switched memory B, median (IQR) cells**	2 (0-6)	21.5 (9.2-35)	9 (4.5-72)	10-80	<0.0001 (****)	0.0003 (***)
**$CD21^{low}$ B cells, median (IQR)**	5 (3-9)	5 (3-10.2)	6 (3.7-7.2)	3-35	>0.9999 (ns)	>0.9999 (ns)
**NK cells, median (IQR)**	147 (85-242)	266.5 (174.5-393.8)	216 (126.8-840)	90-600	<0.0001 (****)	0.1275 (ns)
**T cells, median (IQR)**	984.5 (629-1,425)	1,109 (813.5-1,518)	963 (724.5-1423)	800-2,500	0.3758 (ns)	>0.9999 (ns)
**$CD4^+$ T cells, median (IQR)**	472 (295-739)	655 (433-907)	561 (405.5-967.8)	500-1,500	0.0052 (**)	0.4472 (ns)
**$CD4^+$ T follicular helper cells, median (IQR)**	59.5 (27.7-99.2)	55 (24-95.5)	68 (36.1-112)	50-150	>0.9999 (ns)	>0.9999 (ns)
**Naïve $CD4^+$ T cells, median (IQR)**	215 (104-426)	350 (256-559)	262 (161.8-464)	400-1,000	0.0011 (**)	>0.9999 (ns)
**Memory $CD4^+$ T cells, median (IQR)**	205 (132.8-341)	231 (156.3-336.5)	231 (144.8-445)	300-700	>0.9999 (ns)	>0.9999 (ns)
**$CD8^+$ T cells, median (IQR)**	369 (206-658)	386 (207.5-594)	341 (226.8-662.3)	200-800	>0.9999 (ns)	>0.9999 (ns)

CVID, common variable immunodeficiency; IQR, interquartile range
[1] n.s. not significant; p < 0.05 *; p < 0.01 **; p < 0.001 ***; p < 0.0001 ****
